# Spatial–temporal evolution and influencing factors of urban–rural economic circulation in China’s agricultural areas: A case study of Jianghan Plain

**DOI:** 10.1371/journal.pone.0313355

**Published:** 2025-07-09

**Authors:** Xiaoyue Li

**Affiliations:** School of Tourism, The Center of Targeted Poverty Alleviation and Rural Revitalization, Xinyang Normal University, Xinyang, China; Institute of Earth and Environment, Chinese Academy of Sciences, CHINA

## Abstract

Urban–rural economic circulation (UREC) is an important part of the domestic economic circulation and an important element in the construction of the new development paradigm of “dual circulation” in China today. Currently, the uneven development of UREC in China threatens the sustainable development of the urban–rural economy. Quantitative research on UREC is lacking in the academic community. As a result, research at the national level cannot easily provide guidance in agricultural regions. Therefore, theoretical and case-based empirical research on UREC must be carried out urgently. This study proposes a theoretical framework and a measurement index system for UREC. It uses the Jianghan Plain, a traditional agricultural growing area in China, as a case study. It also uses the entropy method and stepwise regression analysis to study the spatiotemporal evolution of UREC and the influencing factors from 2013 to 2020. Results show that, from 2013 to 2020, the UREC index of the Jianghan Plain grew year by year, and the spatial difference increased. Regional administrative power and urban–rural socioeconomic development levels had a significant impact on UREC in the Jianghan Plain, with general public budget expenditures having the greatest positive impact.

## Introduction

Accelerating the construction of a new development paradigm with domestic circulation as a mainstay and the mutual reinforcement of dual domestic and overseas circulation is a major strategic measure for adapting to the new stage of domestic development and new changes in the international environment [1–3]. “Dual circulation” can be categorized into different levels, such as inter-country, inter-regional, and urban and rural levels. As stated by the President of China, General Secretary Xi Jinping, at the second of the five plenary sessions of the 19th CPC Central Committee, “urban–rural economic circulation is an important part of the domestic circulation and a key factor in ensuring the health of the proportionality of the two circulations on domestic and overseas markets” [4]. As global urbanization and industrialization proceed, economic factors are concentrated in urban areas, and rural areas are declining in many parts of the world [5,6]. This scenario affects the development of urban and rural economies and the sustainable development of regional economies [7–9]. At present, China’s urban–rural economic circulation (UREC) has development problems due to the traditional system of urban–rural division [10,11]. This scenario is reflected in the wide disparity between urban and rural development, the failure to satisfy the production and living needs of urban and rural residents, the dual structure of urban and rural industries, and the poor flow of factors between urban and rural areas. These problems are the concrete manifestations of the uneven development of UREC, which has become one of the shortcomings of domestic circulation in China [12]. Therefore, the spatial and temporal evolution of UREC and its influencing factors must be explored in the context of the construction of China’s dual circulation and the evolution of urban–rural relationships.

A great deal of research on UREC exists, which is scattered among topics such as the demand-supply relationship, the industrial linkages and flows of factors in urban and rural economic activities, and the basis for interaction between urban and rural economies. However, the content of the studies is scattered. At the present level, no high degree of consensus exists on the conceptual connotation of UREC. Systematic and comprehensive research on UREC at the theoretical level is also lacking. The existing studies have mostly centered on policy-oriented research at the national level, and empirical research on specific cases has been slightly insufficient. In the present study, the Jianghan Plain was selected as the research area. It is a traditional agricultural area with relatively lower levels of development than the rest of China at this stage; the spatiotemporal evolution of its UREC and the factors influencing it are typical of agricultural areas. The possible contributions of this study are as follows: First, the UREC in the Jianghan Plain was analyzed as regional economic circulation based on certain urban–rural linkages. Second, the spatiotemporal changes in UREC in the Jianghan Plain were analyzed at the aggregate and sub-dimensional levels, and the factors influencing these changes were further investigated. Third, the results demonstrated that the smooth optimization of UREC in the Jianghan Plain can contribute to the development of the general domestic circulation. The findings of this study can provide case references for other regions of agricultural development. The rest of the paper is organized as follows: Section 2 describes the relevant literature; Section 3 reviews the materials and methods; Section 4 presents the results; Section 5 presents the discussion; Section 6 provides conclusions from the research.

## Literature review

The UREC research originated from research on economic circulation and urban–rural relationships. Economic circulation, originally studied from the perspective of economics, began with a study of the French national economic circulation published by François Quesnay (1694-1774) in the book *Tableau Économique*. In terms of research content, studies of economic circulation are rather abundant. Their main content includes the study of the dynamic equilibrium of supply and demand at the macro-level [13,14], the study of the rational development of industrial structures and industrial relations at the meso-level [5,15,16], and the study of the processes of economic activities in which economic factors constantly flow with the changes in the development of the supply–demand relationship and industries at the micro-level [17]. Research methods have gradually become highly diverse with the increase in research content. They include the entropy value method, input–output method, and principal component analysis method, among others. UREC is based on a certain urban–rural relationship as a background. The study of urban–rural relationships evolved from natural sequence theory of Adam Smith, who claimed that rural areas preceded urban areas, followed by the theory of urban–rural co-development, “top–down” urban bias theory, “bottom–up” rural bias theory, and “up–down” theory of urban–rural integration [18,19].

The studies related to UREC include the supply and demand relationship, industrial linkages, factor flows, and the basic study of the interaction between urban and rural economic activities. The supply and demand relationship between urban and rural areas is generated by the differences or complementarities between urban and rural areas geographically [20]. The supply and demand interaction between urban and rural areas is used to meet urban and rural development needs and improve productivity. The cross-penetration of production activities between urban and rural areas leads to the intersection and integration of urban and rural industries [21,22]. Rural-urban economic interaction stems from the differences between urban and rural industrial sectors. Agriculture, which mainly involves planting and breeding, is concentrated in rural areas; industry, which mainly involves manufacturing and related services, is mainly concentrated in urban areas [10]. Factors are fundamental to economic development, and the participation of factors such as labor, land, capital, and technology provides basic raw materials and power for urban and rural development [23]. The research related to the factors of production in the process of UREC mainly includes the dimensions of labor, land, and capital. The mismatch between the supply and demand of factors of production in the process of product production and industrial activities in certain urban and rural areas drives the flow of factors between urban and rural areas [24]. The spatial distance between urban and rural areas cannot be changed [25]. Given the spatial distance between urban and rural areas, the flow of products and factors between urban and rural areas involves flow costs [26,27]. Moreover, the integrated development between urban and rural areas is the basis of the economic circulation between urban and rural areas [28]. Spatial interaction theory, proposed by Ullman, shows that if the spatial distance friction is too large, then an interaction cannot happen even if a complementary supply and demand relationship exists [29]. Related research topics based on the interaction between UREC include urban–rural transportation, information, and market development. Relevant research shows that based on infrastructure construction, the capacity system is upgraded in terms of urban–rural transportation development; thus, urban and rural products can be transported to urban and rural territories and the links between urban and rural areas can be constructed [30]. Information technology plays an important role in UREC, and without adequate communication, there is no way to establish links between urban and rural economic activities cannot exist [31]. Forms of ICT increase virtual and physical connectivity and improve communication infrastructure [32]. Inadequate network connectivity and limited ICTs may hinder the supply and marketing of rural and urban products [33]. In terms of the development of integrated rural-urban markets, productive activities and interactions between rural and urban territories are difficult to generate in the absence of a unified market that mobilizes labor, capital, and other inputs [24].

UREC is the economic circulation of urban and rural territories. Studies on the measurement of dual circulation proliferated after the new development pattern of building dual circulation was proposed in China [34–36]. However, measurement-based research on UREC, which is an important component of the total domestic circulation, is still lacking. Qualitative studies of UREC can provide many insights into its measurement. However, research on UREC, which is an important component of the total domestic circulation, is insufficient. The analysis of the conceptual connotation of UREC has not yet been unified, and the research on quantitative measurement needs to be further deepened. In terms of the research on the conceptual understanding of UREC, Shengwei Tu pointed out that UREC is a process involving the flow of factors, industrial coupling, and matching supply and demand between two heterogeneous spaces in urban and rural areas [37]. Shuyi Feng considered that UREC involves a harmonious spatial pattern of urban–rural economic development, equal exchange, and a two-way flow of factors between urban and rural areas, as well as the equalization of urban and rural public services and infrastructures [12]. Li Xiaoyue argues that UREC refers to the process of urban–rural interaction between certain urban and rural areas, which is manifested by supply and demand relations at the macro level, with industrial linkages as the main medium, factor flows as the micro content, and cyclic carriers as the basic support [38].

**Fig 1:**
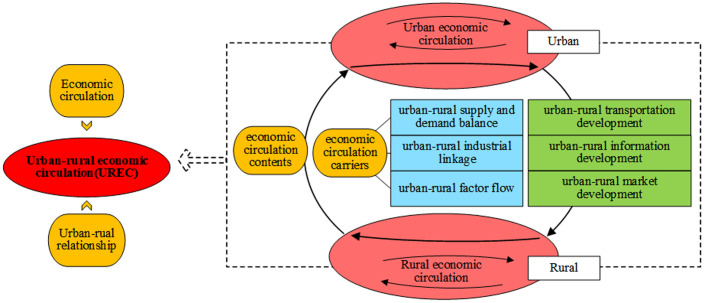
The theoretical framework.

On the basis of these studies, we argue that UREC includes two aspects: the content of economic circulation and the carriers of economic circulation between urban and rural areas (Fig 1). ① The main content of UREC is the economic circulation between cities and rural areas. Urban and rural production and development have differences in terms of natural conditions, resource attributes, geographical location, and socioeconomic foundations. Rural areas are mainly engaged in agricultural production, and their basic function is to provide raw materials and food security for cities [39]. The cities are mainly engaged in non-agricultural industries, the production of non-agricultural products, and the provision of services and employment [40], which radiate into and drive the development of rural areas [41–43]. The functional differentiation between urban and rural areas triggers the heterogeneity of supply and demand in the urban and rural production process. The fulfillment of urban and rural supply and demand requires industry as a medium, whereas the development of industry requires the input of factors of production. Thus, the circulation content of UREC mainly consists of three aspects: the balance of supply and demand, industrial linkages, and the flow of factors between urban and rural areas. ② The carriers of economic circulation in UREC are the spaces containing economic activities carried out between urban and rural areas. These carriers connect urban and rural spaces and are an important prerequisite for the development of economic activities between urban and rural areas [44]. The development level of these carriers determines the possibility of UREC taking place. In particular, these carriers include urban–rural transportation development, urban–rural information development, and urban–rural market development [45,46].

UREC is influenced by many factors. The relevant studies on influencing factors include natural environment elements, socio-economic elements, and regional development policies. In the studies on the influence of the natural environment, Wei Pan et al. emphasized the influence of topographic topography on UREC and, argued that topographic topography has an impact on the immovable aspects of UREC, including urban–rural land elements and urban–rural interconnections in UREC, such as urban–rural market infrastructures and public service facilities [47]. Haller et al. studied the impact of altitude on urban–rural interactions and concluded that the higher the altitude is, the more pronounced the phenomenon of urban–rural separation is; they also found that urban–rural interactions in mountainous areas are characterized by special characteristics [48]. In the study of socio-economic impacts, urbanization, industrialization, and globalization are considered the driving factors of the socio-economic development dimension of UREC. The development of urbanization accelerates the flow of economic factors between urban and rural areas. The development of industrialization affects the content and level of industrial development in urban and rural areas, thereby affecting the interaction process between urban and rural industries [32,49,50]. The development of globalization has led to interregional economic, social and cultural interconnections and integration, which accelerate the linkages between urban and rural areas and the international economy, and the process of urban–rural economic interactions [51–53]. In the study of the impact of regional development policies, government-led national or regional macro-policy factors constitute the external environment of UREC [54]. The policy regimes of urban–rural development and regional development have a significant impact on UREC process [55]. It can hinder or accelerate the process of economic circulation between urban and rural areas [56]. These studies provide useful instructions for studying the influencing factors of UREC.

## Materials and methods

### Study area

**Fig 2:**
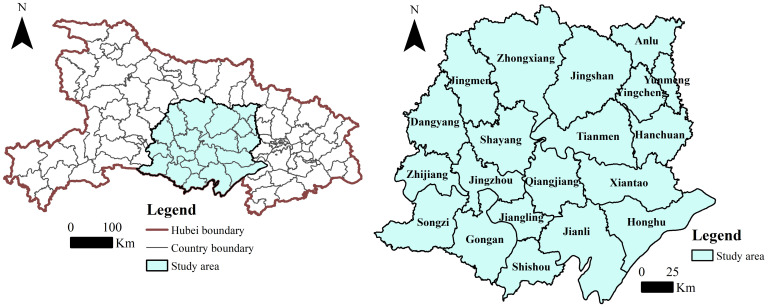
Location of the Jianghan Plain, Hubei Province.

The Jianghan Plain (Fig 2) is a traditional agricultural area in China, and it is relatively underdeveloped at present [57]. Rapid urbanization and industrialization have significantly influenced UREC in the Jianghan Plain, and the evolution of its UREC pattern has characteristics typical of agricultural areas. The Jianghan Plain is located in the south-central part of Hubei Province, at a longitude of 110°20′07′′–137°71′77′′ E and latitude of 32°83′337′′–35°24′172′′N. It comprises the following: the three provincial municipalities of Xiantao, Qianjiang, and Tianmen; the cities of Yunmeng, Yingcheng, Anlu, and Hanchuan in Xiaogan City; the city of Jingzhou and its precincts; the city of Jingmen and its precincts, with 20 county-level administrative units. The land area of the Jianghan Plain is 41,700 km^2^, occupying 22.43% of the land area of Hubei Province. From 2013 to 2020, the population of the Jianghan Plain declined from 15.75 million to 14.17 million, the rural population declined from 8.09 million to 6.34 million, the urbanization rate increased from 48.63% to 55.25%, and the gross domestic product increased from RMB 489.12 billion to RMB 722.16 billion.

### Selection of the measurement indicator for UREC

UREC is the economic circulation between urban and rural areas according to the spatial scale and scope of economic activities [58]. The study of UREC is the study of regional economic circulation based on certain urban–rural relationships. On the basis of the theories related to the economic circulation and urban–rural relationship and the connotation analysis of UREC, Li Xiaoyue [38] and He Peike [59] et al. selected the corresponding measurement indexes based on systematic, scientific, and data-accessible principles.

According to the aforementioned analysis, UREC mainly consists of two aspects: the content of economic circulation and the carriers of economic circulation between urban and rural areas (Table 1). ① Based on the connotation of the economic circulation, the content of economic circulation of UREC is interpreted as the supply–demand balance, industrial linkage and factor flow between urban and rural areas. The supply–demand balance between urban and rural areas is the general target of UREC. Moreover, the overall supply capacity of urban and rural areas is their overall production level, which can be characterized by GDP. The overall demand level of urban and rural areas is their consumption capacity, which is characterized by the total retail sales of consumer goods. The degree of balance in urban–rural supply and demand is closely related to the income gap between urban and rural residents, which can be characterized by the urban–rural income ratio. The urban–rural industrial linkage is a concrete manifestation of the content of economic circulation in UREC [60,61]. The level and stage of development of urban and rural industries can be characterized by the night light index and the ratio of secondary and tertiary sector GDP to primary sector GDP [62]. The interaction between urban and rural industries can be characterized by a binary comparison coefficient [9]. The flow of urban and rural factors is the micro-content of UREC. Economic input factors are mainly considered in the form of urban–rural factors, which mainly include labor, land, and capital [63]. In terms of labor force flows in urban and rural areas, the ratio of the number of non-agricultural workers in rural areas is considered. The flow of land factors between cities and rural areas is essentially the transformation of regional land-use types between agricultural land and urban construction land, which is characterized by the ratio of the area of urban construction land to that of urban–rural land. The flow of capital between cities and rural areas is characterized by the loan balances of financial institutions per unit of GDP. ② Given the physical distance between urban and rural areas in the process of interconnections and interactions [41], the content of the economic circulation activities between urban and rural areas needs carriers to connect the urban and rural areas [64,65]. The development of urban–rural transportation integration refers to the degree of difficulty in overcoming geographic distance in the process of UREC for products and factors to be transported and moved between urban and rural areas. Moreover, it can be reflected by the state of urban and rural transportation infrastructure construction in terms of index selection for the carrier of economic circulation, with the length and density of roads in urban and rural areas being the main measurement dimensions. The length and density of roads within a certain urban and rural area were selected as two indicators to reflect the overall level of urban and rural transportation infrastructure construction. The development of urban–rural information integration refers to the level of difficulty in communicating and exchanging information on product supply and demand, industrial development, and factor allocation between urban and rural areas. The development of urban and rural information is characterized by the Baidu index, which reflects the degree of intensity of information flows between urban and rural areas [66]. The larger the value is, the higher the level of information development in urban and rural areas is. Information infrastructure construction is also important. The number of telecommunication business halls is selected to describe the overall construction level of urban and rural information infrastructure. The development of urban–rural market integration refers to difficulties in economic activities, such as the trading of products and factors in urban and rural areas and the development of industries across urban and rural areas. The development of urban–rural markets is characterized by the urban–rural market scale indicator, which is calculated as the sum of the products of urban and rural incomes and the population size [67]. The form in which the market exists includes offline markets, such as wholesale markets, shopping centers, and virtual markets in online networks. Given the reality of this study, the economic factor circulation flow between urban and rural areas relying on markets mainly involves physical-based markets. Therefore, the number of market POI points within a certain urban and rural region is selected to reflect the development of urban and rural markets.

**Table 1:** Index system for the evaluation of UREC.

First-level indicators	Second-level indicators	Specific indicators	Index property	Weight
Content of economic circulation	Urban–rural supply and demand balance	GDP	+	0.107
		Total retail sales of consumer goods	−	0.114
		Urban–rural income ratio	+	0.096
	Urban–rural industrial linkage	Night light index	+	0.122
		The ratio of secondary and tertiary sector GDP to primary sector GDP	+	0.116
		Binary comparison coefficient	+	0.113
	Urban–rural factor flow	The ratio of the number of non-agricultural workers in rural areas	+	0.095
		The ratio of urban construction area to urban–rural area	+	0.120
		The loan balances of financial institutions per unit of GDP	+	0.118
Carriers of economic circulation	Urban–rural transportation development	Length of the roads	+	0.155
		Density of the roads	+	0.150
	Urban–rural information development	The Baidu index	+	0.175
		The number of telecommunication business halls	+	0.174
	Urban–rural market development	The urban–rural market scale	+	0.164
		The number of market POI points		0.181

### Data sources

The availability of data was considered, 2013–2020 was selected as the study period. The spatial data of the Jianghan Plain used in this study came from the Earth System Science Data Sharing Platform (www.geodata.cn). The socioeconomic statistics were obtained from the “China County Statistical Yearbook (County and Municipal Volume)”, “Hubei Statistical Yearbook”, “Hubei Rural Statistical Yearbook”, and county and municipal statistical yearbooks and almanacs. Rare missing data were added through linear interpolation. The Baidu index data were obtained from the official website of the Baidu index (https://index.baidu.com/v2/index.html#/), and the night light index was obtained from the EANTLI night light dataset from 2000 to 2020. The telecom business hall and market POI point data were obtained from the Baidu Map Open Platform (https://1bs.baidu.com).

## Methods

### Entropy method

The entropy method is based on using the information provided by the observed values of each index to determine weights, thereby making it an objective assignment method. The interference of human factors is effectively eliminated, and the objectivity and scientific validity of the evaluation results are ensured. In this study, the entropy method was used to obtain the index weights of the circulation content index and circulation carrier index. The calculation steps are as follows:

All of the raw data were standardized using the standardization method of polar deviation. The specific steps are shown in Formula 1.


$$\text{Positive indicators: }Z_{ij}=\frac{X_{ij}-X_{min}}{X_{max}-X_{min}}$$

$$\text{Negative index: }Z_{ij}=\frac{X_{max}-X_{ij}}{X_{max}-X_{min}}$$ (1)

Here, $Z_{ij}$ is a standardized indicator value; i and j represent, the ith research unit and the jth indicator, respectively; $X_{ij}$ is the original value; $X_{max}$ and $X_{min}$ are the maximum and minimum values of each indicator, respectively.

The indicator weights were calculated. The specific steps are shown in Formulas 2 and 3, assuming that n research units and m indicators exist.


$$P_{ij}=\frac{Z_{ij}}{\sum_{i=1}^{\text{n}}Z_{ij}}$$ (2)

$$E_{j}=-\left(\frac{1}{\ln n}\right)\overset{\text{n}}{\underset{i=1}{\sum }}(P_{ij}\ln P_{ij})$$ (3)

$$W_{j}=\frac{1-E_{j}}{\sum_{j=1}^{\text{m}}\left(1-E_{j}\right)}$$ (4)

Here, $P_{ij}$ is the proportion of the ith research unit to all research units; $E_{j}$ and $W_{j}$ are the entropy value and weight of index j.

The circulation content index and circulation carrier index of UREC in each research unit in each particular year were calculated.


$$A_{i}=\overset{m}{\underset{j=1}{\sum }}W_{j}Z_{ij}$$ (5)

Here, $A_{i}$ is the circulation content index or circulation carrier index.

### Comprehensive evaluation model

The circulation content index and circulation carrier index of UREC in the Jianghan Plain were calculated separately using the weighted summation method. The circulation content index and the circulation carrier index are equally important for UREC. Thus, the overall UREC index was the average value of the circulation content index and the circulation carrier index (S1 File).

### Stepwise regression analysis

In this study, the stepwise regression analysis method was selected to investigate the analysis of the influencing factors of UREC. Stepwise regression analysis is a common multiple regression analysis method often used to establish the optimal regression model. All the independent variables that have a significant impact on the dependent variable are retained, and the independent variables that have no significant impact on the dependent variable are eliminated. Multiple stepwise regression analysis is a method for establishing the optimal multiple linear regression equation. Stepwise regression analysis was performed using SPSS 25.0 software, with the UREC index as the dependent variable and the influencing factors as independent variables.

## Results

### Spatial–temporal evolution of UREC in the Jianghan Plain

#### Characteristics of the temporal evolution.

**Fig 3:**
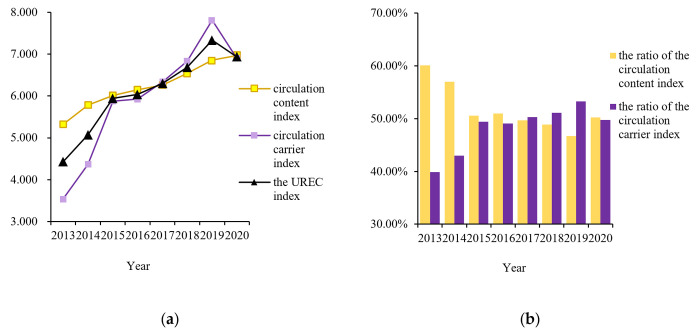
Temporal evolution of the UREC index in 2013-2020.

Fig 3 shows that the UREC index, the circulation content index, and the circulation carrier index in the Jianghan Plain indicated an upward trend as a whole from 2013 to 2020, with the scores of each index, increasing from 0.221, 5.321, and 3.532 in 2013 to 6.969, 6.889, and 6.895, respectively, and the yearly growth rates increasing at average rates of 8.09%, 4.43%, and 13.60%, respectively. Within the study period, the ratio of the circulation content index of UREC in the Jianghan Plain fluctuated and decreased, whereas the ratio of the circulation carrier index fluctuated and increased. The percentage of the circular carrier index increased from 39.90% in 2013 to 49.74% in 2020.

**Fig 4:**
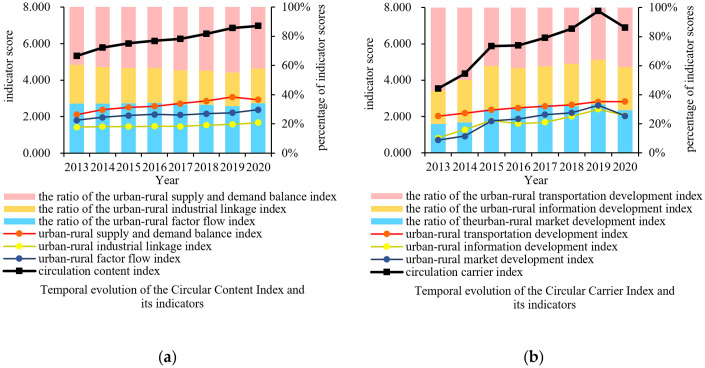
Temporal evolution of indicators of UREC from 2013 to 2020.

In terms of the evolution of the circulation content index of UREC from 2013 to 2020 (Fig 4), the urban–rural supply and demand balance, industrial linkage, and factor flow indexes showed overall growth trends, with the scores of each index increasing from 2.104, 1.417, and 1.799 to 2.922, 1.671, and 2.375, respectively, and the average yearly growth rates being 5.55%, 2.56%, and 4.58%, respectively. ① The urban–rural supply and demand balance index had the highest score, the highest growth rate, and the highest proportion among the three indexes. The urban–rural supply and demand balance index in 2020 decreased compared with that in 2019. This outcome may have been due to the outbreak of the coronavirus disease (COVID-19) at the end of 2019, which led to a decline in urban–rural supply and demand. ② The urban–rural industrial linkage index had the lowest score, showing slow growth from 2013 to 2017 and fast growth after 2018. However, accounted for the lowest percentage among the three indexes during the study period. ③ The urban–rural factor flow index showed a generally high growth rate in the study period, and the proportion of this index slowly increased.

In terms of the evolution of the indexes of circulation carriers in UREC from 2013 to 2020 (Fig 4), the indexes of urban–rural transportation, information, and market development showed a general trend of growth, with the scores of each index increasing from 2.020, 0.812, and 0.701 to 2.818, 2.048, and 2.029, respectively, and the average yearly growth rates being 5.65%, 21.76%, and 27.08%, respectively. The following were observed during the study period: ① The urban and rural transportation development index score showed yearly growth. Compared with the urban–rural information and market development indexes, this index grew relatively slowly, resulting in a decrease in its percentage from 57.18% to 40.87%. ② The urban and rural information development index showed fluctuations and increased as a whole, with its percentage increasing from 22.99% to 29.71%. ③ The urban and rural market development index showed rapid growth in 2013–2015, slow growth in 2016–2019, and a declining trend in 2020. This finding demonstrated that the scale of urban and rural markets needs to be further improved.

#### Characteristics of spatial evolution.

**Fig 5:**
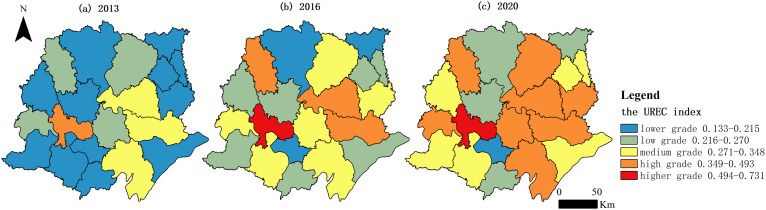
Spatial evolution of the UREC index.

The natural breakpoint method was used to present the spatial patterns accurately, the. This method involves classifying the scores into five levels, from high to low. The spatial distribution of UREC in the Jianghan Plain was mapped u sing the ArcGIS software (Eari Corporation, Redlands, CA, USA) by spatially correlating the scores of each research unit in the form of vectors with each research unit. Fig 5 shows the overall spatial distribution of the UREC scores for each county-level administrative unit in the Jianghan Plain. In terms of spatial distribution, no higher grade areas could be observed in 2013, and only Jingzhou was a high grade area. The remaining 80% of the regions were low grade areas or lower grade areas, except for Xiantao, Tianmen and Jianli, which were medium grade areas. In 2016, only one higher grade area existed, which was Jingzhou, and Jingmen, Xiantao and Tianmen were high grade areas. The number of medium grade areas was six, the number of low grade areas increased to seven, and the number of lower grade areas decreased to three. In 2020, Jingzhou remained to be the only higher grade area. The number of high grade areas increased from three in 2016 to eight in 2020. The number of medium grade areas is the same as in 2016. The low grade areas are Zhongxiang, Yunmeng, Shayang and Shishou. Only one lower grade area existed, which was Jiangling. The spatial differences had an expanding trend as the level of UREC improved. In the study period, the spatial differences in the UREC in the Jianghan Plain were large, and they showed an increasing trend. In 2013, the maximum UREC value (Jingzhou) was 0.396 and the minimum (Jiangling) was 0.133, thereby making the maximum 2.977 times the minimum. In 2015, the maximum UREC value (Jingzhou) was 0.588, and the minimum (Jiangling) was 0.162, making the maximum 3.630 times the minimum. In 2020, the maximum UREC value (Jingzhou) was 0.731, and the minimum (Jiangling) was 0.209, making the maximum 3.498 times the minimum. The spatial coefficients of variation in the UREC in the Jianghan Plain in 2013, 2016, and 2020 were 0.281, 0.309, and 0.348,respectively.

**Fig 6:**
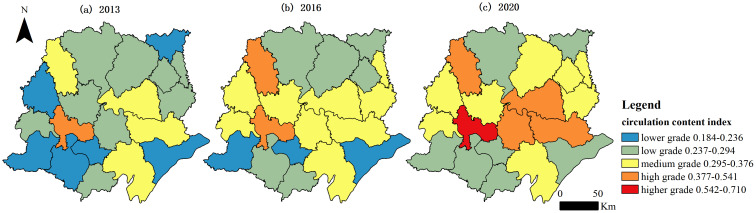
Spatial evolution of the indicators of the circulation content index.

**Fig 7:**
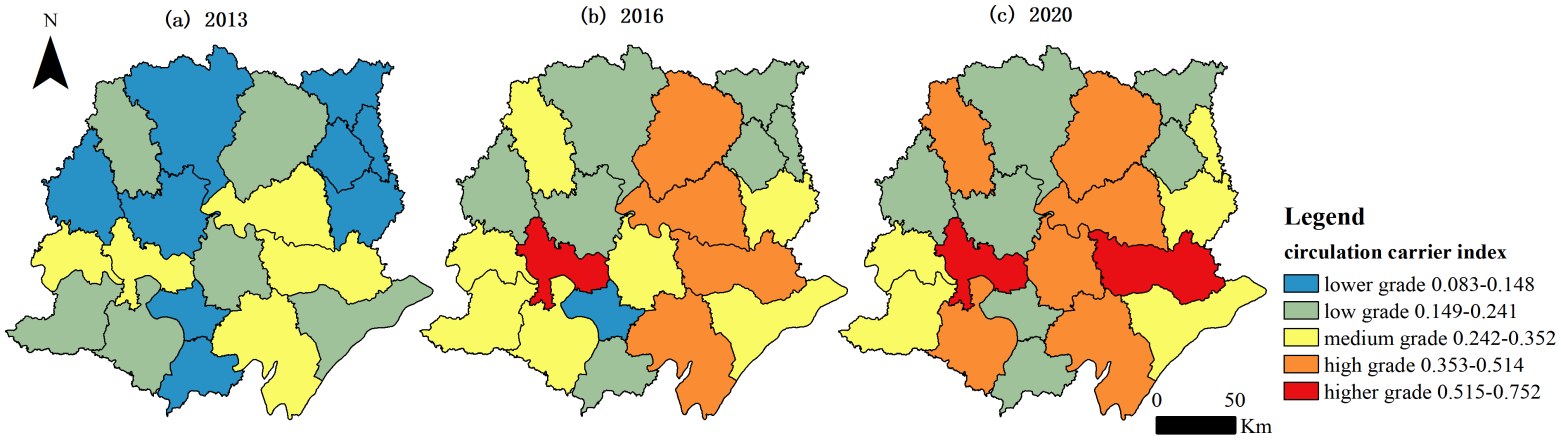
Spatial evolution of the indicators of the circulation carrier index.

Figs 6 and 7 show the spatial distribution of the circulation content and circulation carriers in UREC in each county-level administrative unit in the Jianghan Plain. The circulation content index evolved from a spatial distribution pattern dominated by lower and low grades in 2013 to a spatial pattern with a dispersed distribution of high to low grades in 2020. The spatial variation in the circulation content index increased during the study period. The coefficient of variation in the circular content index increased from 0.211 in 2013 to 0.250 in 2016 and then to 0.302 in 2020. The spatial distribution of the circulation carrier index and that of the UREC index had a general similarity. The spatial distribution was dominated by lower grades and low grades in 2013. Moreover, twelve low-grade areas still existed in 2020. Two higher-grade areas, six high-grade areas, and five medium-grade areas existed in terms of the circulation carrier index in the Jianghan Plain in 2020. The results showed that the spatial distribution of the indicators of circular content and circular carriers displayed a trend of evolution toward higher grades in the spatial distribution and no lower grade areas existed at the end of the study period. Moreover, the circulation carrier index exhibited a great spatial variation during the study period, with a coefficient of variation of 0.440 in 2013, which decreased to 0.413 in 2016 but then increased to 0.431 in 2020.

### Analysis of the influencing factors of the UREC in the Jianghan Plain

#### Selection of influencing factors.

UREC is influenced by natural environmental elements, socio-economic elements and regional development policies. The difference considered in the natural environmental conditions of the study units within the Jianghan Plain was not much. Therefore, the focus was placed on the influence of socio-economic elements and regional development policies. The socioeconomic development levels of regions and urban and rural areas in the context of regional government policies are important factors that influence the level of UREC [68]. Regional development policies and the level of regional socio-economic development are external influences on UREC, and the level of urban and rural socio-economic development are internal influences on UREC. In terms of external influences, policies related to regional development, including urban and rural development, affect urban–rural relations, thereby affecting UREC. Regional socio-economic development is the regional context for the development of UREC, and the economic basis for urban–rural development. In terms of internal influences, the level of urban socio-economic development and the level of rural socio-economic development directly affect the level of UREC.

Therefore, the influence factors of UREC were selected in this study from four aspects: regional development policies, regional socioeconomic development, urban socioeconomic development, and rural socioeconomic development. The influence factor indicators were selected based on comprehensiveness, representativeness, and data availability. ① Government policies focus on the effects of the external forces of financial resources and policy favoritism on UREC [40]. For the regional government policy, local general public budget expenditures (X1) and the number of new enterprises in the region (X2) were selected. The public financial output reflects the government’s active intervention in the quality of economic development by investing in public goods and services [69]. This study used the local general public budget expenditures as an indicator to characterize the level of government investment in regional economic development. ② Regional socioeconomic development mainly includes the regional population size and the status of regional industrial development [70]. Regional socioeconomic development was characterized by the population size and industrial sophistication; the regional population was used to characterize the population size (X3), and the output value of secondary and tertiary industries per unit of GDP (X4) was chosen to characterize the level of industrial sophistication in the region. ③ Urban socioeconomic development mainly includes the level of urbanization and the development of urban industrialization and globalization [71]. The urban socioeconomic development was characterized by the level of urbanization, industrialization and globalization. The regional proportion of urban population (X5), the total industrial output value (X6), and the exports of international trade (X7) were selected as representative indicators. ④ Rural socioeconomic development mainly includes the level of agricultural development and its degree of modernization [72]. Rural socioeconomic development was characterized by the output value of agriculture and the degree of modernization of agriculture. Moreover, the output value of agriculture (X8) and the total power of agricultural machinery per unit of sown area (X9) were selected as indicators.

#### Analysis of the regression results.

Stepwise regression analyses were conducted to understand the influences of various factors on the level of UREC in the Jianghan Plain, with the nine indicators described above as independent variables and the UREC index as the dependent variable (Table 2). Five factors were left in the final model as predictor variables: local general public budget expenditures (X1), regional population (X3), the regional proportion of urban population (X5), total industrial output value (X6), and output value of agriculture (X8). The adjusted R^2^ value of the model was 0.898, indicating that these six factors can explain 89.8% of the variation in the dependent variable. The Variance Inflation Factor (VIF) value of each influencing factor was below 5.0, indicating that the model had no redundant variables and that no multicollinearity existed among the factors. The Durbin–Watson (D-W) value was 1.868, indicating that the sample data had autocorrelation and that the fitting result was good. The model passed the F-test (F =  10.760; p =  0.001 <  0.05), indicating that the model was effective.

**Table 2:** Results of the analysis of the influencing factors of UREC based on stepwise regression.

Variable	Regression Coefficient	t	p Value	VIF
Local general public budget expenditures (X1)	0.645	14.522	0.000	3.067
The regional proportion of urban population (X5)	0.223	5.742	0.000	2.339
The regional population (X3)	0.135	0.135	0.000	2.132
The output value of agriculture (X8)	0.126	3.995	0.000	1.550
The total industrial output value (X6)	−0.059	−3.280	0.001	1.016
Sample size	160			
Adjusted R^2^	0.898			
F value	10.760			
Dependent variable: the UREC index				
D-W value: 1.868				

According to the absolute value of the standardization coefficient, we can observe the influence of each influencing factor on the level of the UREC in the Jianghan Plain and the degree of importance. They are presented as follows in order of importance: X1 > X5 > X3 > X8 > X6. X1, X5, X3, and X8 were positively correlated with the level of UREC, whereas X6 was negatively correlated with it. The influencing factors of the UREC in the Jianghan Plain were related to the regional administrative power and the level of socioeconomic development of urban and rural areas. The study shows that the influencing factors of the UREC in the Jianghan Plain were significantly related to the regional administrative power and the level of urban and rural socio-economic development. The local general public budget expenditure (X1) was the primary factor influencing the UREC in the Jianghan Plain. Moreover, the influence of local general public budget expenditures and the number of new enterprises in the region on the level of UREC was positive. This finding implies that the more capital a regional government invests and the stronger the policy support is, the higher the level of UREC is. Capital investment by local governments not only has a positive impact on the development of urban–rural integration but also has a positive and significant impact on the development of UREC. The regional proportion of the urban population (X5) was a secondary factor affecting the UREC in the Jianghan Plain. It indicates that the level of urbanization had a positive impact on UREC. The higher the level of urbanization is, the greater the population transferred from rural to urban areas is. Moreover, all kinds of production factors are concentrated in urban areas because of market mechanisms. Urbanization is the engine of regional economic development, driving regional socio-economic development and consequently UREC. Therefore, urban sectors receive high revenue, which can be used to drive and promote the economic development of rural areas and the healthy circulation of the urban and rural economies. The regional population (X3) was one of the factors that positively affected the UREC in the Jianghan Plain as a general factor. The number and scale of people, as the actors of economic activities, mirror the degree of economic vitality. The larger the population scale is, the more dynamic the economic development is, the closer the economic interaction between urban and rural areas is, and the higher the level of UREC is. The output value of agriculture (X8) was the fourth major factor in the UREC of the Jianghan Plain. Agriculture was the most basic industry for regional development, and the development of the agricultural industry had a positive impact on UREC. This finding indicates that the enhancement of UREC requires a solid foundation for the development of the agricultural industry. Aithough the total industrial output value (X6) had the minimum impact on the UREC in the Jianghan Plain, it showed a negative impact. The development of industrialization requires inputs of high levels of factors of production, and such resources are limited in traditional agricultural growing areas. The higher the level of urban industrialization is, the more factors of production are absorbed by urban industrialization. This scenario leads to a lack of corresponding factor inputs for the development of the rural economy and society and affects UREC. As a result, it would have a negative impact on UREC. This finding indicates that although industrialization provides impetus for regional and urban development, focusing on the development of rural areas in parallel with the development of industrialization is also important.

## Discussion

UREC is an important part of the national economy. The level of UREC reflects the state of its development and is closely related to the sustainable development of urban and rural economies. Studying the spatiotemporal evolution of UREC and the factors influencing its practice in different regions is important. China is committed to building a new development paradigm of “dual circulation”, and scholars have deeply studied domestic and international circulation. However, little direct research on the economic circulation between urban and rural areas, which is an important component of it, has been conducted. The study of UREC is particularly important in the Jianghan Plain, which is an agricultural area with relatively low levels of economic development compared with the rest of China.

This study mainly discusses UREC through the content and carriers of circulation between urban and rural areas in the context of the construction of dual circulation and the evolution of urban–rural relationships. Twenty county-level administrative units in the Jianghan Plain were selected as the study area, and a UREC measurement index system was constructed from the aspects of the urban–rural circulation content and circulation carriers. Finally, the UREC index, the circulation content index, and the circulation carrier index in the Jianghan Plain from 2013 to 2020 were analyzed. The UREC index increased yearly during the study period. However, the growth rate slowed down after 2016. The circulation carrier index increased yearly and had a good development trend. However, the growth of the circulation content index slowed down after 2016 and was affected by the COVID-19 outbreak at the end of 2019. Then, the circulation content index declined in 2020. The score of the urban–rural industrial linkage index was the lowest, illustrating that the low level of urban–rural industrial linkage led to a slowdown in the development of the circulation content in UREC. The circulation carriers in UREC had a very low level of development at the beginning of the study period. However, the urban–rural circulation carrier index grew at a faster rate because of the continuous practice of the national policy of urban–rural integration. In 2017 and 2018, the economic circulation carrier index was larger than the circulation content index. This finding shows that the national development of urban–rural integration played an important role in supporting the healthy development of economic circulation between urban and rural areas. Furthermore, the phenomenon of widening differences in UREC between the regions of the Jianghan Plain is a notable issue. This observation indicates that although the overall level of UREC improved, the development gap between regions increased. At the end of the study period, Jingzhou had the largest UREC value of 0.731, whereas Jiangling had the smallest at 0.209. Moreover, the coefficient of variation between regions at the end of the study period was 0.348, which was 23.84% higher than that at the beginning of the study period.

According to the existing studies on topics related to UREC, natural environmental factors, socio-economic factors, and regional development policies are the main influencing factors. Based on the study of factors influencing the urban-rural economic cycle in the Jianghan Plain, a traditional agricultural area in China, this study confirmed the important influence of regional development policies and socio-economic factors on UREC. The study results indicate the positive influence of government financial inputs, regional population size, urbanization level, and agricultural development level onUREC in the Jianghan Plain and the weak negative influence of industrialization development on UREC.

These results reflect that the government plays an important role in the urban-rural economic cycle in the Jianghan Plain, that size of the regional population is the driving force for economic development, the concentration of the population in towns and cities is an important manifestation of the strengthening of urban-rural interactions, and agriculture and the countryside are the most basic guarantee for urban-rural economic interactions. The results also indicate that in the future, paying consideration pay more attention to the development of the rural areas and the urban-rural integration and development while promoting the socio-economic development of the region and urban and rural areas will be necessary. This finding reflects the important role of the government in the UREC of the Jianghan Plain, with the regional population size being the driving force of economic development, the concentration of the population in the urban areas being an important manifestation of the strengthening of urban-rural interactions, and the agriculture and the rural areas being the most basic guarantee of urban-rural economic interaction. Thus, in the future, paying considerable attention to the development of rural areas and the urban-rural integration development while promoting regional and urban-rural socio-economic development will be necessary.

This study had some limitations. First, the conceptual definition of UREC has not been unified in academic circles, and research on the theoretical connotations of UREC needs to be further deepened. This study argued that UREC is regional economic circulation based on a certain urban–rural relationship, and UREC was interpreted in terms of circulation content and circulation carriers. Second, this study was limited by the difficulty of obtaining indicator data because of the dynamics and complexity of the spatiotemporal evolution of UREC. Only some quantifiable indicators were selected for the study of UREC in the Jianghan Plain. Optimizing the evaluation index system for UREC, analyzing the influencing factors of the development of UREC, and expanding the case study to other similar regions in China are important issues that need to be further considered and deepened in the future.

## Conclusions

Based on the content and carrier of UREC, this study analyzed the comprehensive connotations of UREC, proposed a theoretical framework and measurement index system, and enriched the theoretical and empirical research. This study used the UREC of Jianghan Plain, a traditional agricultural area in China, as an example to enrich the research cases of UREC. The study reveals that from 2013 to 2020, the UREC index of the Jianghan Plain increased by 56.60%, the growth trend of urban and rural circulation carriers fluctuated greatly, and the content of UREC showed a yearly growth trend. Moreover, the score of the UREC index in the Jianghan Plain showed an increasing spatial difference every year. Local government policies, regional and urban-rural socio-economic development dominated the changes in UREC. Increasing the financial investment in regional development and improving the level of urban and rural socio-economic development can improve and optimize UREC, thereby providing inspiration for the sustainable development of the urban and rural economy.

## Supporting information

S1 Fig The theoretical framework. (TIF)

S2 Fig Location of the Jianghan Plain, Hubei Province. (TIF)

S3 Fig Temporal evolution of the UREC index in 2013-2020. (TIF)

S4 Fig Temporal evolution of indicators of UREC from 2013 to 2020. (TIF)

S5 Fig Spatial evolution of the UREC index. (TIF)

S6 Fig Spatial evolution of the indicators of the circulation content index. (TIF)

S7 Fig Spatial evolution of the indicators of the circulation carrier index. (TIF)

S1 File The UREC index. (XLSX)

S1 Table Index system for the evaluation of UREC. (TIF)

S2 Table Results of the analysis of the influencing factors of UREC based on stepwise regression. (TIF)
