# Correction: Spatial–temporal evolution and influencing factors of urban–rural economic circulation in China’s agricultural areas: A case study of Jianghan Plain

**DOI:** 10.1371/journal.pone.0335557

**Published:** 2025-10-27

**Authors:** Xiaoyue Li

S1 Table is uploaded incorrectly. Please see the correct S1 Table here.

## Supporting information

S1 Table Index system for the evaluation of UREC. (TIF)
