# Supplementary figures and images for: Spatial–temporal evolution and influencing factors of urban–rural economic circulation in China’s agricultural areas: A case study of Jianghan Plain
Source: PLoS One. 2025 Jul 9;20(7):e0313355. doi: 10.1371/journal.pone.0313355 (PMC12240347; doi:10.1371/journal.pone.0313355)

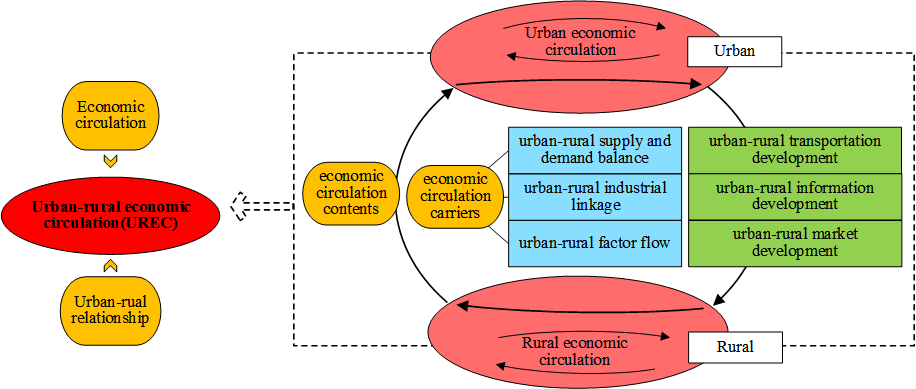

Supplement: S1 Fig — (TIF) [file pone.0313355.s001.tif]

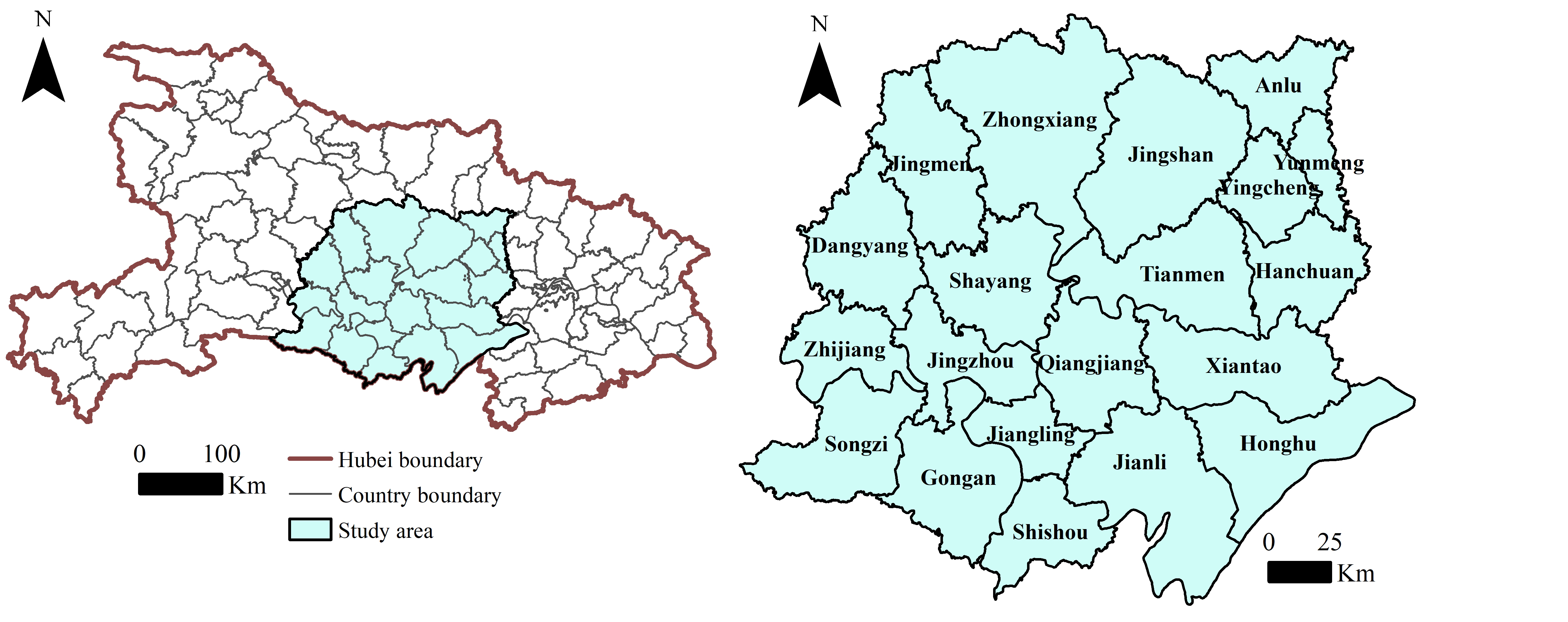

Supplement: S2 Fig — (TIF) [file pone.0313355.s002.tif]

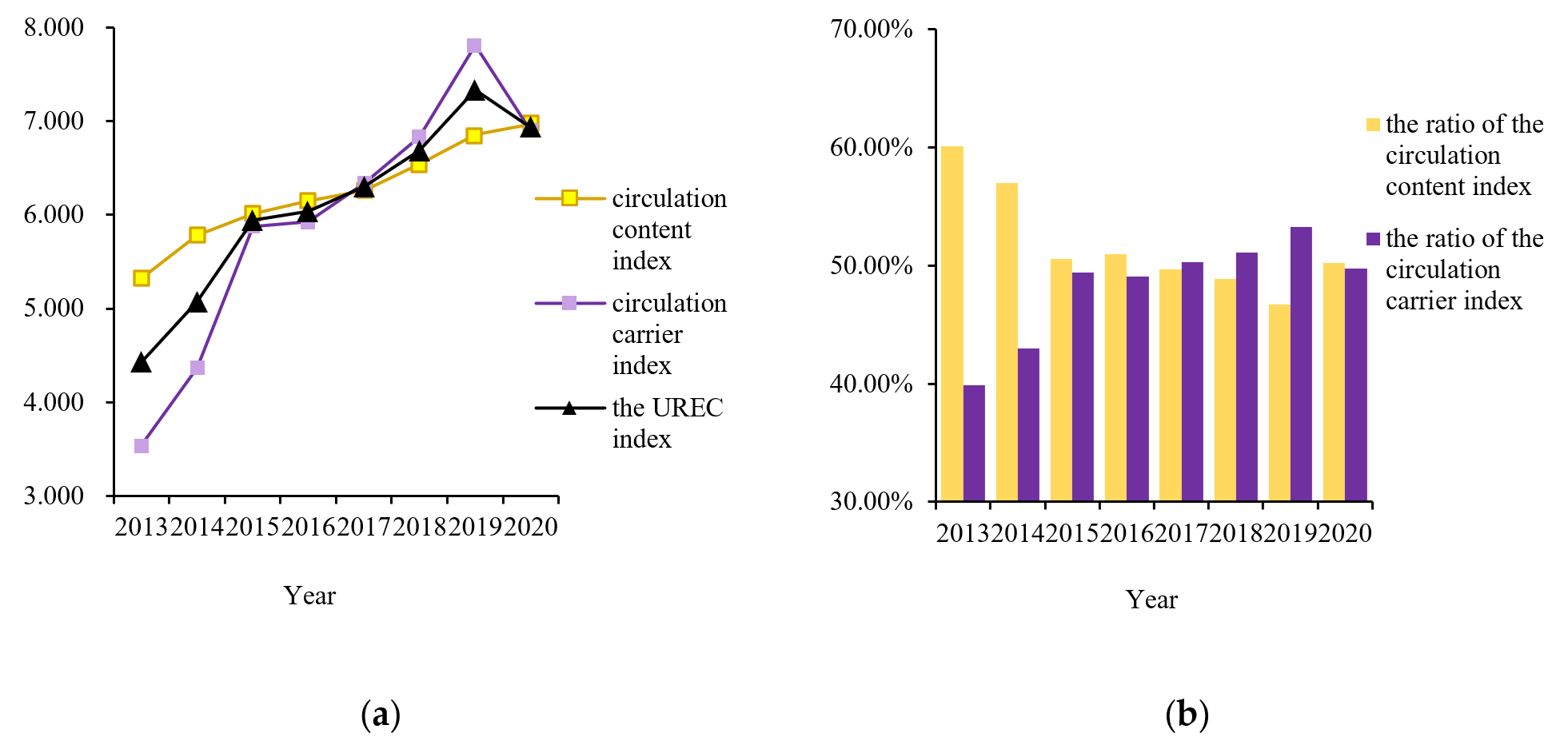

Supplement: S3 Fig — (TIF) [file pone.0313355.s003.tif]

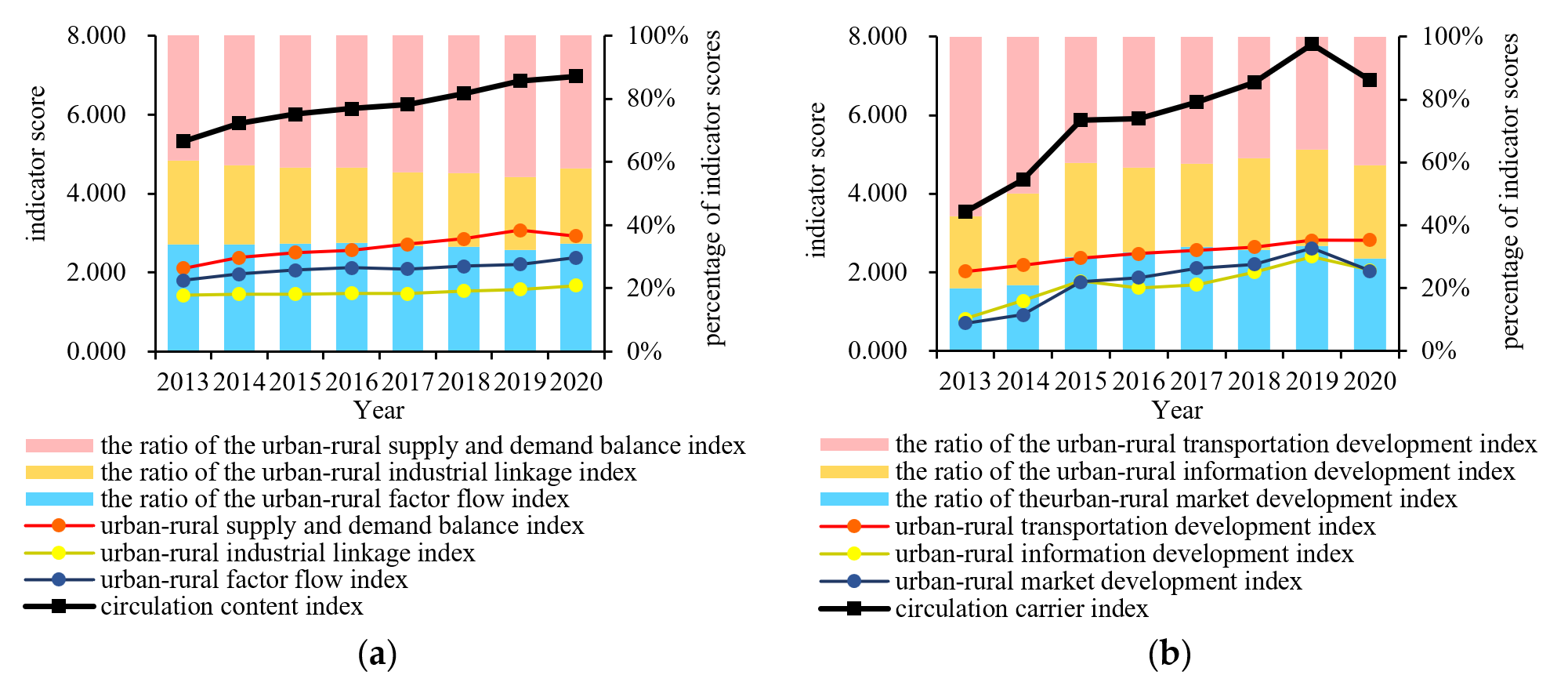

Supplement: S4 Fig — (TIF) [file pone.0313355.s004.tif]

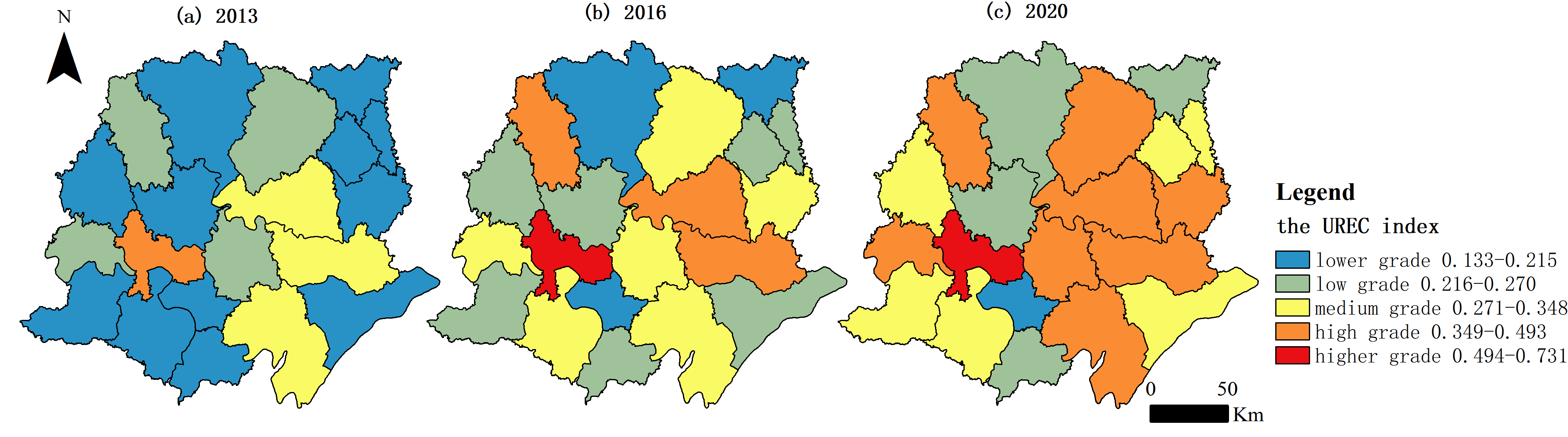

Supplement: S5 Fig — (TIF) [file pone.0313355.s005.tif]

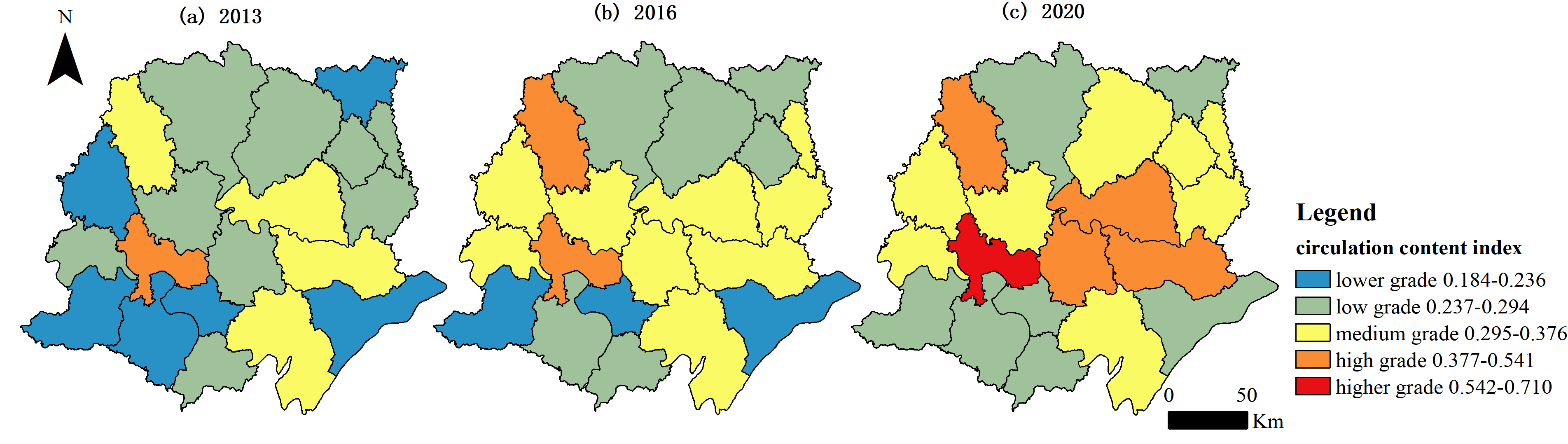

Supplement: S6 Fig — (TIF) [file pone.0313355.s006.tif]

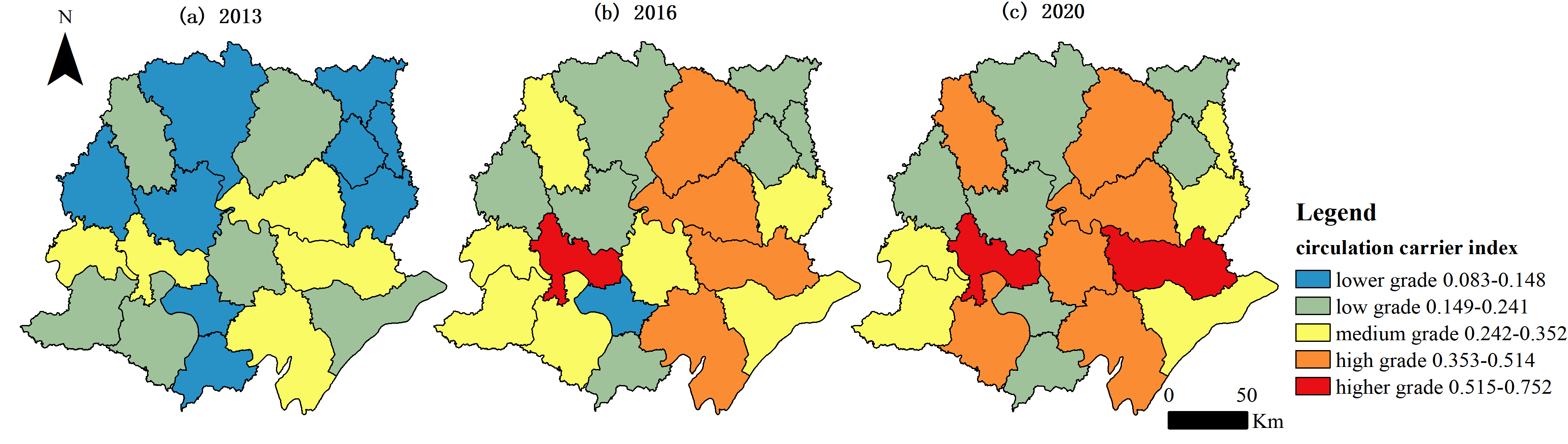

Supplement: S7 Fig — (TIF) [file pone.0313355.s007.tif]

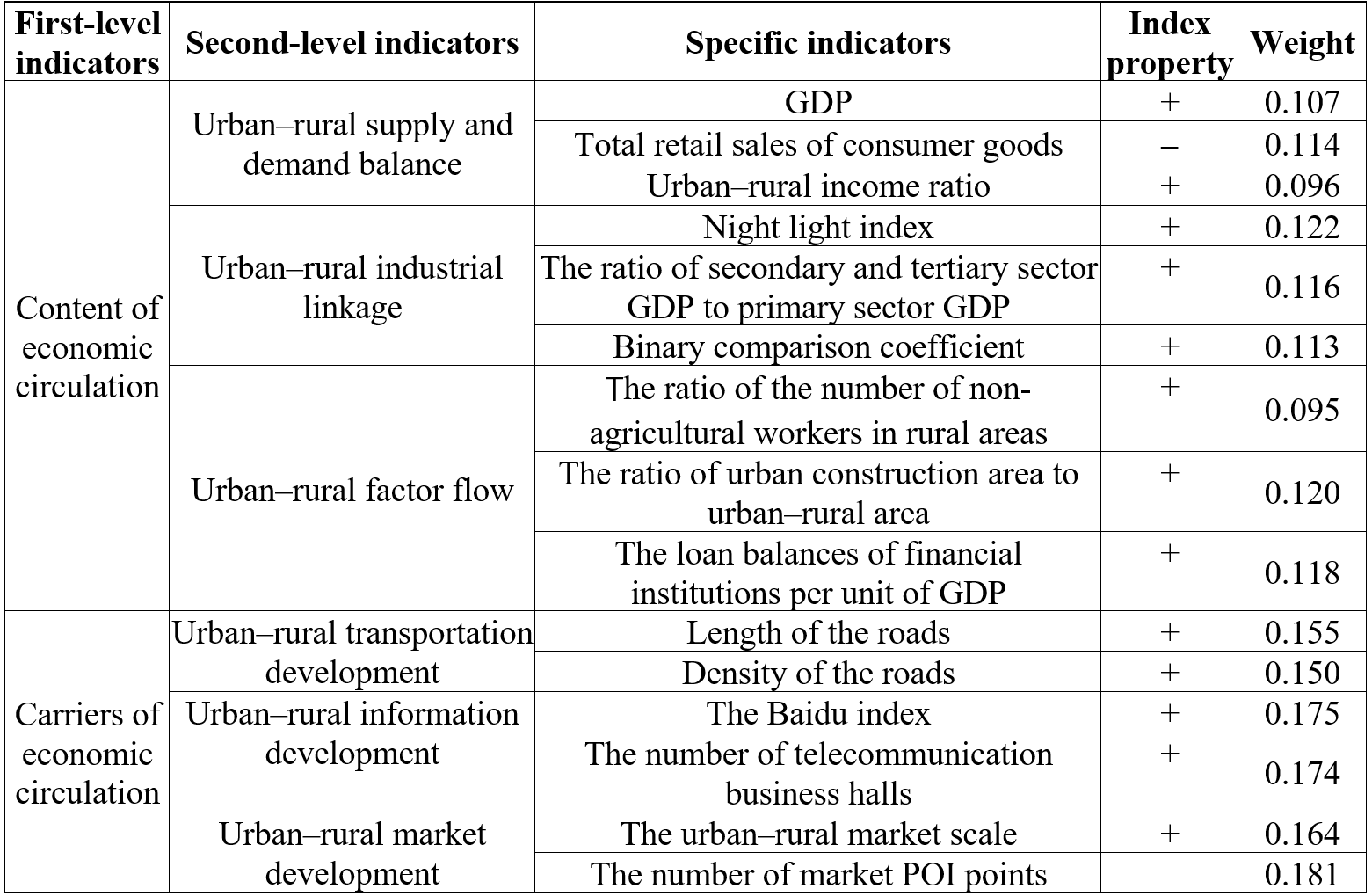

Supplement: S1 Table — (TIF) [file pone.0313355.s009.tif]

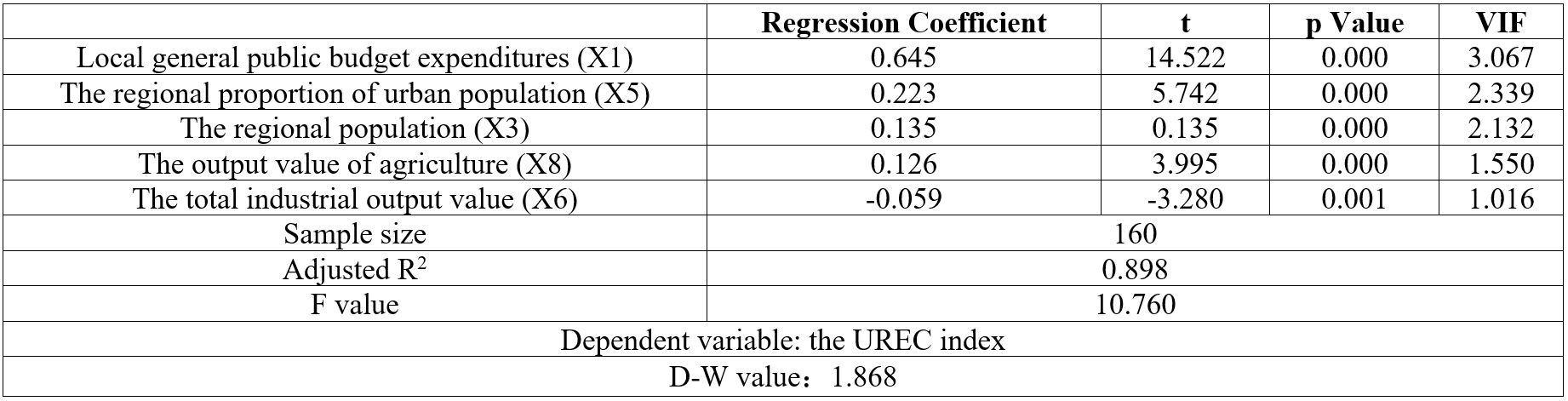

Supplement: S2 Table — (TIF) [file pone.0313355.s010.tif]
