# Supplementary figures and images for: Correction: Spatial–temporal evolution and influencing factors of urban–rural economic circulation in China’s agricultural areas: A case study of Jianghan Plain
Source: PLoS One. 2025 Oct 27;20(10):e0335557. doi: 10.1371/journal.pone.0335557 (PMC12558471; doi:10.1371/journal.pone.0335557)

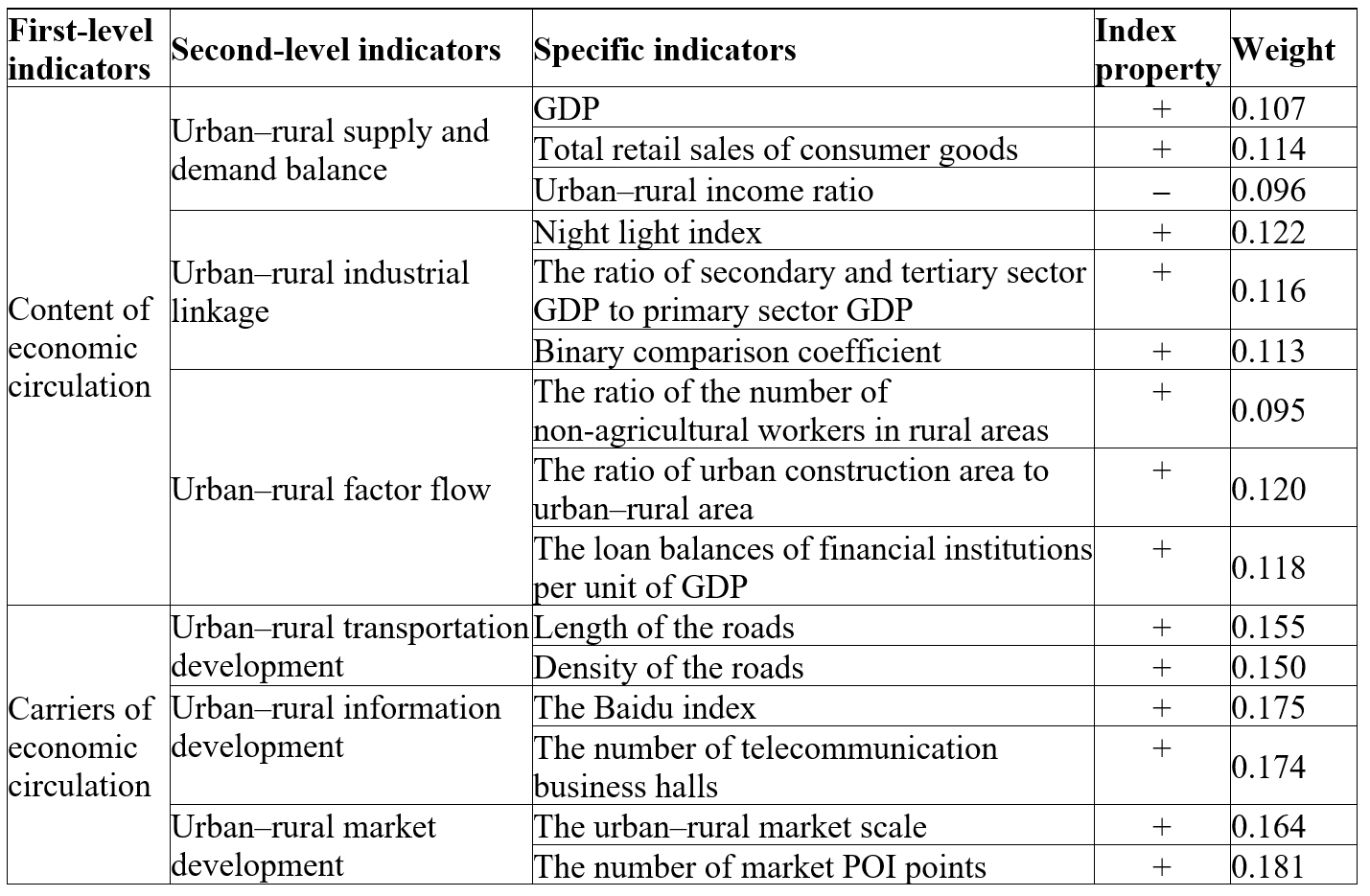

Supplement: S1 Table — (TIF) [file pone.0335557.s001.tif]
